# Computational and Experimental Prediction of Human C-Type Lectin Receptor Druggability

**DOI:** 10.3389/fimmu.2014.00323

**Published:** 2014-07-10

**Authors:** Jonas Aretz, Eike-Christian Wamhoff, Jonas Hanske, Dario Heymann, Christoph Rademacher

**Affiliations:** ^1^Department of Biomolecular Systems, Max Planck Institute of Colloids and Interfaces, Potsdam, Germany; ^2^Department of Biology, Chemistry, and Pharmacy, Freie Universität Berlin, Berlin, Germany

**Keywords:** C-type lectin receptors, druggability, inhibitor, DC-SIGN, langerin, MCL, fragment screening, NMR screening

## Abstract

Mammalian C-type lectin receptors (CTLRS) are involved in many aspects of immune cell regulation such as pathogen recognition, clearance of apoptotic bodies, and lymphocyte homing. Despite a great interest in modulating CTLR recognition of carbohydrates, the number of specific molecular probes is limited. To this end, we predicted the druggability of a panel of 22 CTLRs using DoGSiteScorer. The computed druggability scores of most structures were low, characterizing this family as either challenging or even undruggable. To further explore these findings, we employed a fluorine-based nuclear magnetic resonance screening of fragment mixtures against DC-SIGN, a receptor of pharmacological interest. To our surprise, we found many fragment hits associated with the carbohydrate recognition site (hit rate = 13.5%). A surface plasmon resonance-based follow-up assay confirmed 18 of these fragments (47%) and equilibrium dissociation constants were determined. Encouraged by these findings we expanded our experimental druggability prediction to Langerin and MCL and found medium to high hit rates as well, being 15.7 and 10.0%, respectively. Our results highlight limitations of current *in silico* approaches to druggability assessment, in particular, with regard to carbohydrate-binding proteins. In sum, our data indicate that small molecule ligands for a larger panel of CTLRs can be developed.

## Introduction

Glycans are present in a large diversity on cell surfaces and are essential in many aspects of life such as embryonic development, cell–cell communication, and regulation of the immune system (1). In particular, our understanding of the role of glycans in immunobiology has grown significantly during the last decades. Three major families of secreted or membrane-bound lectins recognize carbohydrates. Complementary to other receptors of the innate and adaptive immune system, Galectins, Siglecs, and C-type lectins shape the response to incoming signals (2, 3). Among many other processes, they are involved in pathogen recognition and killing, antigen processing, and tumor progression (2, 4, 5).

Mammalian C-type lectin receptors (CTLRs) represent a large family of lectins, which is subdivided into 17 groups based on their phylogenetic relationships and domain structure (6). CTLRs are present in a variety of tissues and the glycan specificity of receptors present on cells of the innate immune system has been studied extensively. For example, they function as homing receptors on leukocytes as well as pattern recognition receptors (2, 3, 7). A particularly well-studied pattern recognition receptor is the dendritic cell-specific intercellular adhesion molecules-3-grabbing non-integrin (DC-SIGN) (8, 9). This CTLR is expressed on dendritic cells and macrophages and is involved in the recognition of a large array of pathogens such as *Mycobacterium tuberculosis, Leishmania*, HCV, Ebola, and HIV (3, 10–15). It was demonstrated that DC-SIGN promotes HIV *trans*-infection of T cells and has since then drawn attention as a therapeutic target in anti-viral therapy (10, 16, 17).

Aside from interference with pathogen recognition, leukocyte homing has been a target for small molecule inhibition of CTLR function. To this end, Selectins, a group of three CTLRs, have been in the focus as anti-inflammatory drug targets since the mid-90s (18). Only recently, the glycomimetic GMI-1070 has entered clinical trials for the treatment of sickle cell anemia (19). Likewise, agonistic CTLR ligands hold promise to serve as adjuvants for immune stimulation (20). However, despite increasing interest in CTLRs as pharmacological targets, there is only a limited set of small molecule agonists or antagonists available (17). Partially, this can be attributed to the limited success of previous attempts to find lead structures from classical drug discovery campaigns.

All CTLRs share a C-type lectin domain (CTLD) that has a conserved fold with a characteristic double-loop stabilized by two disulfide bridges (7, 21). This domain is often referred to as carbohydrate recognition domain (CRD) for those CTLRs involved in glycan binding. Additional domains are frequently present and in particular, heptad-repeats and collagen-like neck domains promote oligomerization, resulting in high avidity glycan binding. In transmembrane CTLRs, CRD, and neck domain are referred to as extracellular domain (ECD). Canonical carbohydrate recognition is mediated by a calcium ion and although there are four Ca^2+^ binding sites, only the second site (Ca^2+^-2) is described to be involved in coordinating glycans (21). While Ca^2+^-4 has not been associated with carbohydrate binding, positive cooperative effects are observed between the other sites (22, 23). Not all potential Ca^2+^ sites are occupied in every CTLD, which reflects the fine-tuned physiological role of this interaction. For endocytic CTLRs the pH sensitivity of the heptad-repeat neck formation and Ca^2+^ coordination as well as active Ca^2+^ export from the endosome are major contributors to endosomal ligand release (23, 24). Some CTLRs bind carbohydrates in a Ca^2+^-independent, non-canonical binding site with Dectin-1 being the prime example (25). All CRDs share a carbohydrate recognition site that is largely flat and hydrophilic. This is a consequence of glycans being highly hydrophilic themselves (17, 26). Hence, binders are also often hydrophilic and do not suffice the requirements for orally available drugs (27).

Whether a protein is a suitable candidate for drug development is of major concern during the drug discovery process. Considering the expenses involved in the development of a pharmacologically active small molecule, target selection has to be done carefully (28). The modulation of a suitable drug target with a rule of five compliant molecules should result in a therapeutic effect (29). The term druggability, however, refers to the ability of a protein to bind a drug-like ligand with high affinity and specificity (29–31). Furthermore, this interaction has to result in a modulation of the protein function. Importantly, a high druggability does not infer the protein being a good drug target. The latter definition includes a therapeutic effect induced by small molecule binding (32, 33). Methods to assess the druggability of a target protein have become good predictors prior to starting a drug discovery campaign, as low scores are indicators for a high failure rate during later stages of the project (30, 33).

The availability of structural information enables computational assessment of druggability. Limited resources are required and many computational tools have been developed to deduce druggability scores from crystallographic information (34, 35). In a two-step process, pockets on the protein surface are first identified and then scored (28, 32, 34, 36). Large sets of proteins can be analyzed and predictions have been found to correlate well with experimental data (31, 34, 37, 38). To the best of our knowledge, there are only two studies on the computational druggability assessments of glycan-binding proteins, both reporting low scores (39, 40).

Experimental assessment of target druggability can be pursued even in the absence of structural information. For this, screening of drug-like molecules in a high-throughput screening format can be performed. Previous reports on micromolar inhibitors of DC-SIGN resulting from a screening campaign highlight the success of this approach (41, 42). Alternatively, a diverse library of fragments of drug-like molecules is screened against the target. The molecular weight of these fragments ranges between 150 and 300 Da. Estimates propose that 1000 fragments can cover a similar chemical space as 10 trillion drug-sized molecules would (33). This in turn allows applying smaller libraries to test the druggability of a candidate protein (31, 33). The low complexity of fragments increases their likelihood of binding a receptor and consequently hit rates of 5–15% are regularly observed for druggable targets (31, 37, 43).

Small molecule fragments have low affinities with dissociation constants in the upper micro- to lower millimolar range. Hence, sensitive biophysical techniques are necessary to monitor this interaction and nuclear magnetic resonance (NMR) spectroscopy has established itself as one of the major techniques used for fragment screening (31, 33, 37–39, 44, 45). In particular, hit rates from NMR-based screenings have proven to be reliable measures of druggability (31, 37, 44). In ligand-observed NMR, mixtures of fragments are screened against a target and changes in NMR observables such as chemical shift, line width, and signal intensity upon binding allow hit identification. Notably, deconvolution of the fragment mixtures is not necessary. The use of fluorine atoms in drug-like fragments has proven to be instrumental (38, 46). As fluorine is rare in biological samples, ^19^F NMR spectra of fragment cocktails are not perturbed by background resonances. Moreover, the fluorine spin is highly susceptible to changes in its chemical environment and allows sensitive identification of hits.

To predict the druggability of human CTLRs, we compiled a set of 22 crystal structures and analyzed it by applying computational methods. We then chose DC-SIGN and conducted experimental fragment screening to compare these findings. Low druggability scores derived *in silico* did not match the moderate to high fragment hit rates during experimental evaluation. Hence, we expanded our screening by two additional CTLRs, namely Langerin and MCL and discovered similarly high experimental druggability estimates. Taken together, our results highlight the limitations of *in silico* druggability prediction for CTLRs while our fragment screening present promising grounds for inhibitor design against this family.

## Materials and Methods

### Structure-based multiple sequence alignment and consensus structure

The scope of structural data on human CTLRs was assessed using the protein family (Pfam) database (accession code: PF00059) (47). Natural killer (NK) cell lectin-like receptors were treated as a closely related, yet physiologically distinct subfamily according to the Pfam annotation and were not included in the analysis. Furthermore, CTLRs crystallized as a domain swap dimer, namely blood dendritic cell antigen 2 (BDCA-2) and mannose receptor (MR), were omitted (48, 49). Murine Dectin-1 was included in the selection as it has an unusual Ca^2+^-independent carbohydrate-binding mode and no structural information of the human ortholog is available (25). All structures considered for analysis are listed (Table 1). If available, a structure in complex with a carbohydrate ligand was selected. Prior to the calculations, all structures were trimmed down to the respective CRD domain as inferred from the Pfam domain definition. A structure-based multiple sequence alignment was performed in molecular operating environment (MOE) (50). Pairwise root mean square deviation (RMSD) values were determined for all pairs of C_α_ atoms unless a gap was found in one of the compared sequences. Next, a phylogenetic analysis based on the pairwise sequence similarities was conducted in R (51, 52). Hierarchical clustering was performed based on the Manhattan metric and via the complete linkage criterion. To complement the phylogenetic analysis, MOE was used to predict a consensus structure of all CRDs. During model construction, up to 20 gaps and RMSD values of C_α_ up to 10 Å were allowed for a single position in the multiple sequence alignment.

**Table 1:** **List of analyzed CTLR structures**.

Receptor	PDB code	Domain	Oligomerization	Reference
ASGPR	1DV8	CRD	Monomer	Meier et al. (53)
CD23a	2H2T	CRD	Monomer	Wurzburg et al. (54)
Clec1b	2C6U	CRD	Monomer	Watson et al. (55)
Clec5a	2YHF	CRD	Monomer	Watson et al. (56)
Clec9a	3VPP	CRD	Monomer	Zhang et al. (57)
DC-SIGN	2XR6	CRD	Monomer	Unpublished
DC-SIGNR	1K9J	CRD	Monomer	Feinberg et al. (58)
mDectin-1	2CL8	CRD	Monomer	Brown et al. (25)
E-Selectin	1ESL	CRD–EGF	Monomer	Graves et al. (59)
EMBP	1H8U	CRD	Monomer	Swaminathan et al. (60)
Langerin	3P5F	CRD	Monomer	Feinberg et al. (61)
	3KQG	ECD	Trimer	Feinberg et al. (62)
L-Selectin	3CFW	CRD–EGF	Monomer	Unpublished
LOX-1	1YPO	ECD	Dimer	Park et al. (63)
MBP-C	1HUP	ECD	Trimer	Sheriff et al. (64)
MCL	3WHD	ECD	Monomer	Furukawa et al. (65)
Mincle	3WH3	ECD	Monomer	Furukawa et al. (65)
P-Selectin	1G1S	CRD–EGF	Monomer	Somers et al. (66)
Reg3a	1UV0	CRD	Monomer	Abergel et al. (67)
Reg1a	1QDD	CRD	Monomer	Gerbaud et al. (68)
SCARA4	2OX8	CRD	Monomer	Feinberg et al. (48)
SP-D	3IKN	ECD	Trimer	Shrive et al. (69)
Tetranectin	1HTN	CRD	Monomer	Nielsen et al. (70)
	1TN3	ECD	Trimer	Kastrup et al. (71)

### Binding site prediction and *in silico* druggability assessment

Initially, CTLR structures were superposed in MOE. For superposition and the subsequent druggability assessment, physiologically relevant oligomerization states were assumed (Table 1). The EGF domains of Selectin structures were removed. The resulting files served as input data for binding site prediction with DoGSite (72). The predicted binding sites were mapped on the structure and classified into four categories following the reported nomenclature of secondary structure elements and Ca^2+^ binding sites (21): (i) Ca^2+^-2-binding sites, (ii) Ca^2+^-associated binding sites in long loop, (iii) Ca^2+^-independent carbohydrate-binding sites, and (iv) other binding sites. A binding site was assigned to category (i) if the Ca^2+^-2 ion was part of the predicted binding site. For category (ii), the criteria were less restrictive and all binding sites with residues within a 6 Å radius of either Ca^2+^-1, 2, or 3 were included (Figure S1 in Supplementary Material). Binding sites in category (iii) are located in close proximity to the experimentally determined Ca^2+^-independent carbohydrate-binding site. The druggability of all binding sites was scored with DoGSiteScorer (73). Finally, category (i), (ii), or (iii) binding sites that displayed the highest score for a receptor were selected and this selection served to determine a mean druggability score for the analyzed CTLRs.

### Cloning

Codon optimized genes for DC-SIGN and human Langerin for expression in *E. coli* were purchased from Life Technologies (Carlsbad, CA, USA) and GenScript (Piscataway, NJ, USA), respectively. The DC-SIGN gene included a C-terminal TEV (tobacco etch virus) cleavage site and a Strep-tag II for affinity purification. The ECD and CRD, ranging from amino acids 62 to 404 and 250 to 404 (Figure S3 in Supplementary Material), respectively, were cloned into a pUC19 vector using primers including a T7 promoter and ribosomal binding site (RBS) upstream of the gene (Table 2). Human Langerin truncated ECD, ranging from amino acids 148 to 328, was cloned with a C-terminal TEV cleavage site and a Strep-tag II into a pET32a expression vector (EMD Millipore, Billerica, MA, USA). The MCL gene was obtained from the DNASU Plasmid Repository (HsCD00507041, Arizona State University, Phoenix, AZ, USA) and the ECD was cloned into a pUC19 vector already carrying a Strep-tag II, a T7 promoter and an RBS. For MCL ECD, amino acids ranging from 61 to 215 were used (65).

**Table 2:** **Primer sequences used for cloning**.

Primer	Sequence
DC-SIGN ECD F	GCCGCCTCTAGAGAGTAATACGACTCACTATAGGGACTAGAGAAAGAGGAGAAAACTAGATGGC-CAAAGTTCCGAGCAGCATT
DC-SIGN CRD F	GCCGCCTCTAGAGGAGTAATACGACTCACTATAGGGACTAGAGAAAGAGGAGAAAACTAGATGGCT-GAACGTCTGTGTCATCCGTG
Langerin F	GGTGGTCATATGGCC-TCGACGCTGAATGCCCAGATTCCGG
Langerin R	ACCACC-AAGCTTTTATTTTTCAAACTGCGGATG
MCL F	GGCGGCGCCGGC-CATGCAAAGCTCAAATGCAT
MCL R	GCCGCCCTGCAG-GTTCAATGTTGTTCCAGGTA

### Protein expression and purification

All growth media or chemicals used for protein expression and purification were purchased from Carl Roth (Karlsruhe, Germany) if not stated otherwise. Proteins were expressed insoluble in *E. coli* BL21(DE3) (New England Biolabs, Ipswich, MA, USA) or KRX (Promega, Fitchburg, WI, USA). Precultures were grown in 50 mL Luria–Bertani (LB) medium supplemented with 100 mg L^−1^ carbenicillin for DC-SIGN and MCL expression or 35 mg L^−1^ kanamycin for Langerin expression at 37°C in 250 mL baffled shaking flasks at 220 rpm shaking frequency. The precultures of DC-SIGN and MCL were centrifuged (3,000 × *g*, 10 min, 4°C), the supernatant was discarded, and the sediment was resuspended in 500 mL LB medium supplemented with 50 mg L^−1^ carbenicillin. The cells were afterwards cultivated at 37°C in 2.5 L baffled shaking flasks at 220 rpm shaking frequency. Protein expression was induced with 1 mM IPTG (isopropyl β-d-1-thiogalactopyranoside) at OD_600_ = 0.4–0.6 for additional 4 h at 37°C. The preculture of Langerin trECD was diluted directly to OD_600_ = 0.1 into 500 mL of LB medium supplemented with 35 mg L^−1^ kanamycin, 0.01% d-glucose, and 0.05% l-rhamnose for autoinduction of expression. Bacteria were harvested (4,000 × *g*, 20 min, 4°C), frozen, and resuspended in lysis buffer (50 mM Tris–HCl, pH 7.5, 10 mM magnesium chloride, 0.1% Triton X-100, 4 mg lysozyme [Sigma-Aldrich, St. Louis, MO, USA) and 500 U DNaseI (Applichem, Darmstadt, Germany) per gram of wet biomass] and incubated on ice for 4 h. Inclusion bodies were harvested by centrifugation (10,000 × *g*, 10 min, 4°C) and washed thrice with 20 mL washing buffer (50 mM Tris–HCl, pH 8.0, 4 M urea, 500 mM sodium chloride, 1 mM EDTA) to remove soluble proteins.

For DC-SIGN ECD and Langerin ECD purification, the washed inclusion bodies were resuspended and denatured in 40 mL denaturation buffer (6 M guanidine hydrochloride, 100 mM Tris–HCl, pH 8.0, 1 mM DTT) and incubated at 30°C for 1 h or at 4°C over night, following a centrifugation (42,000 × *g*, 1 h, 4°C). The denatured inclusion bodies were slowly diluted threefold with cold binding buffer (TBS, pH 7.8 with 25 mM calcium chloride), supplemented with 1 mM reduced glutathione (GSH, Applichem) and 0.1 mM oxidized glutathione (GSSG, Applichem), and afterwards dialyzed twice against 2 L of this buffer for 24 h at 4°C. After another 2 L dialysis against binding buffer, proteins were purified according to previously published protocols using a mannan agarose affinity chromatography (Sigma-Aldrich) (74).

The washed inclusion bodies of DC-SIGN CRD were resuspended and denatured in 10 mL denaturation buffer and incubated at 30°C for 1 h or at 4°C over night, following a centrifugation (42,000 × *g*, 1 h, 4°C). The solubilized inclusion bodies in the supernatant were refolded by rapid dilution into 50 mL of cold refolding buffer (100 mM Tris–HCl, pH 8.0, 1 M l-arginine, 150 mM sodium chloride, 120 mM sucrose) while stirring at 4°C. After 2 days, protein solution was dialyzed against 2 L of cold buffer W (100 mM Tris–HCl, pH 8.0, 150 mM sodium chloride, 1 mM EDTA) and aggregated protein was removed by centrifugation (42,000 × *g*, 1.5 h, 4°C). The protein was purified using a Streptactin affinity chromatography (IBA, Goettingen, Germany) according to the manufacturer’s instructions.

MCL refolding and purification was performed according to Furukawa and coworkers introducing minor changes in the protocol. Briefly, purification was performed via Streptactin affinity chromatography after dialysis against 2 L of buffer W.

### Fragment library

Fragments were selected from a pool of commercially available compounds from different manufacturers (Sigma-Aldrich, St. Louis, MO, USA; KeyOrganics, Camelford, UK; ACB Blocks, Toronto, ON, Canada; Santa Cruz Biotechnology, Santa Cruz, CA, USA; Vistas-MLab, Moscow, Russia; LifeChemicals, Kyiv, Ukraine; Alfa Aesar, Ward Hill, MA, USA; TCI, Tokyo, Japan; Apollo Scientific, Stockport, UK) using chemoinformatic tools as implemented in MOE and KNIME (75). Only compounds with <23 non-hydrogen atoms and at least one ring were PAINS-filtered and consecutively included in the diversity selection (76). Fragment selection was based on normalized moments of inertia for shape diversity, Tanimoto coefficient (<0.8) using MACCS fingerprint for chemical diversity and scaffold diversity was ensured following definitions given by Murcko and coworkers (77, 78). Maximum pairwise similarities were calculated in MOE using three-point pharmacophore-based fingerprints (GpiDAPH3) as descriptors and Tanimoto coefficient as similarity metric. The same descriptor was used to assess the chemical complexity of the fragments (31).

Fragments were dissolved in d_6_-DMSO (Euriso-Top, Saint-Aubin, France) to 100 mM stock solutions under a nitrogen atmosphere in Matrix plates (Thermo Scientific, Waltham, MA, USA) followed by shaking at room temperature for 18 h at 140 rpm. Fragments were stored at −20°C. Next, each fragment was dissolved under nitrogen atmosphere at 1 mM in 500 μL 10 mM deuterated phosphate buffer, pH 7.0, containing 50 μM d_4_-TSP [(3-(trimethylsilyl)-2,2′,3,3′-tetradeuteropropionic acid, Sigma-Aldrich], 50 μM TFA (trifluoroacetic acid, Sigma-Aldrich), and 0.01% sodium azide (Carl Roth). A ^19^F and ^1^H NMR spectrum of each fragment was recorded for quality control. All NMR studies were measured at 298 K in Norell SP5000-7 5 mm tubes (Norell, Landisville, NJ, USA) on a Varian PremiumCOMPACT 600 MHz spectrometer equipped with an oneNMR probe (Agilent, Santa Clara, CA, USA) with TSP and TFA as internal references. All spectra were analyzed in MestReNova 9.0.0 (Mestrelab Research, Santiago de Compostela, Spain) for identity and for solubility in D_2_O of at least 200 μM. Substances, that did not fulfill these quality criteria (17%), were removed from the library. Chemical shifts were used to design 8 screening mixtures consisting of 36 compounds each. A genetic algorithm was used to solve the optimization problem of mixture prediction (unpublished data). Prior to screening, all mixtures were analyzed in ^19^F NMR spectra after 18–24 h incubation at room temperature to ensure stability of the mixtures. Compounds experiencing precipitation or changes in chemical shift were removed from the following experiments. The quality control left 281 compounds (83%) to be prepared in mixtures of 100 μM compound each, 100 μM TFA, 150 mM sodium chloride in 20 mM Tris–HCl, pH 7.8, in 20% D_2_O (Euriso-Top) that were stored at −20°C as aliquots until used.

### NMR screening

All protein samples were prepared at 20 μM of final concentration in 20 mM Tris–HCl, pH 7.8, with 150 mM sodium chloride and 1 mM EDTA and mixed 1:1 with the screening mixture aliquots resulting in a final protein and compound concentration of 10 and 50 μM, respectively, in 500 μL final volume. Fluorine spectra were recorded with a spectral width of 140 ppm and a transmitter offset at −120 ppm, acquiring 128 scans, with an acquisition time of 0.8 and 2 s relaxation time. T_2_-filtered spectra were recorded using a CPMG pulse sequence with a 180° pulse repetition rate of 50 Hz and duration of 1.0 s using same acquisition and relaxation times (79, 80). Two CPMG spectra were recorded per mixture to cover the full spectral width. A spectrum ranging from −50 to −100 ppm and from −100 to −150 ppm was recorded with 96 and 256 scans, respectively. Screening was performed first in the presence and absence of protein including 0.5 mM EDTA. Next, calcium chloride was added to a final concentration of 10 mM and measurements were repeated. All spectra were analyzed for changes in peak intensity and chemical shift. As an additional quality control, frequent hitters identified during unrelated screening campaigns were removed.

### SPR follow-up screening

All surface plasmon resonance (SPR) measurements were performed on a Biacore^®^ T100 (GE Healthcare, Chalfont St. Giles, UK) with a flow-rate of 10 μL min^−1^ using HBS-P buffer [10 mM HEPES (4-(2-hydroxyethyl)-1-piperazineethanesulfonic acid), pH 7.6, 150 mM sodium chloride, 0.05% Tween-20] at 298 K. DC-SIGN ECD was immobilized on a CM7 Series S sensor chip in a density of 3317 RU using 0.2 M EDC (1-ethyl-3-(3-dimethylaminopropyl)carbodiimide, Sigma-Aldrich) and 0.05 M NHS (*N*-hydroxysuccinimide, Merck, Hohenbrunn, Germany) as coupling reagents. The activated surface was saturated with 1 M ethanolamine (Sigma-Aldrich), pH 8.5, after immobilization. The reference flow cell was treated in the same manner without immobilizing protein. Prior to measurements, the solubility of each compound in SPR buffer was determined by recording absorption spectra at different concentrations between 400 and 800 nm in clear 96-well plates (Nalge Nunc International, Penfield, NY, USA) in a SpectraMax M5 plate reader (Molecular Devices, Sunnyvale, CA, USA). During SPR measurements, fragments were injected for 30 s following a dissociation time of 120 s at 10 μL min^−1^ flow-rate omitting regeneration as fast off-rates were observed for all ligands. To estimate the apparent affinity of a compound, at least three dilutions between 0.1 and 1 mM depending on the solubility were run in triplicates, blanking the data against a corresponding DMSO control. A positive control was included during screening to ensure stability of the sensorgrams. A 1:1 binding model was applied for data fitting:

(1) $$\text{RU}=\text{R}{\text{U}}_{\max}\frac{\left[L\right]}{K_{\text{D,app}+\left[L\right]}}$$

with the fragment concentration [*L*], the measured relative response units RU, the apparent dissociation constant *K*_D,app_, and the maximal relative response units RU_max_ using Origin8.6Gpro (OriginLab, Northampton, MA, USA). The maximal relative response units were estimated using:

(2) $$\text{R}{\text{U}}_{\max}=\text{A}\cdot \text{R}{\text{U}}_{\text{immobilized}}\frac{\text{M}{\text{W}}_{\text{compound}}}{\text{M}{\text{W}}_{\text{protein}}}$$

with the immobilization level of protein RU_immobilized_, the molecular weight of the compound and protein MW_compound_ and MW_protein_, respectively, and the remaining activity of the protein on the chip A. The latter was determined to be 0.6 using 4 as positive control (Figure S4 in Supplementary Material). The apparent affinity constant for each compound was determined under two conditions, either in the presence of 0.5 mM EDTA or 2 mM calcium chloride included in the running and sample buffer. Ligand efficiencies (LE) were calculated applying

(3) $$\text{LE}=-\frac{\text{RT}\ln\left(K_{\text{D,app}}\right)}{\text{HA}}$$

using the apparent dissociation constant *K*_D,app_, the temperature T, the gas constant *R*, and the number of non-hydrogen atoms HA (81).

## Results

### Structure-based sequence alignment identifies canonical carbohydrate-binding sites

**Figure 1:**
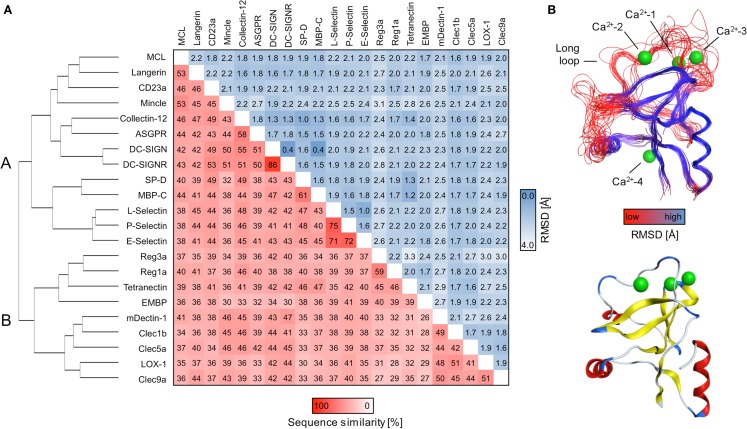
**Sequence alignment and consensus structure of CTLRs**. **(A)** The dendrogram depicts the hierarchical clustering of CRDs based on a structure-based multiple sequence alignment. The two major branches are termed cluster A and B. Pairwise sequence similarities and RMSDs of C_α_ atoms are shown in matrix format. **(B)** Key structural features of the CTLD fold are mapped on the predicted consensus structures (top). The peptide backbone is displayed as a continuous line and colored according to RMSD of C_α_ atoms. The CRD of DC-SIGN (PDB code: 2XR6) is shown for comparison (bottom). The ribbon representation is colored according to secondary structure elements (red: α-helices; yellow: β-strands; blue: loop regions).

A comparative framework between the CTLRs served as the starting point of our druggability prediction. To this end, a structure-based sequence alignment was performed for 22 CRDs (Figure S3 in Supplementary Material). With an average of 41%, the global sequence similarity within the set of receptors is low. It spans a range from 26 to 86% (Figure 1A). A phylogenetic analysis based on this alignment yields a dendrogram that resembles the canonical classifications of CTLRs, in particular with respect to the correct assignment of members of the groups II, III, IV, V, and VII (1). Collectin-12 deviates from this classification, as it is part of the group II cluster. Moreover, Tetranectin and eosinophil major basic protein (EMBP) are the only representatives of group IX and XII used in this study. Both display elevated distances to other branches. EMBP and Tetranectin as well as Clec9a, Lox-1, Clec1b, and Reg1a have been reported to interact with non-carbohydrate ligands and all of these CTLRs were assigned to cluster B. Strikingly, CRDs known to recognize carbohydrates via the Ca^2+^-2-binding site are exclusively present in cluster A (Figure 1A).

### Consensus structure prediction reveals elevated structural variability in the long loop

Contrasting the low global sequence similarity, the overall structure of the CTLD is highly conserved. RMSD values of C_α_ atoms obtained from the structure-based multiple sequence alignment are uniformly low and do not exceed 3.2 Å (Figure 1A). To visualize the conservation of the domain architecture, we calculated a consensus structure (Figure 1B). While the core of the CTLD displays only minor deviations, a higher level of structural variability characterizes the two loop regions. The long loop is of particular interest as it harbors the Ca^2+^-1, -2, and -3 sites and thus plays a fundamental role in Ca^2+^-dependent carbohydrate recognition (21).

### Computational analysis predicts low druggability for the majority of CTLRs

**Figure 2:**
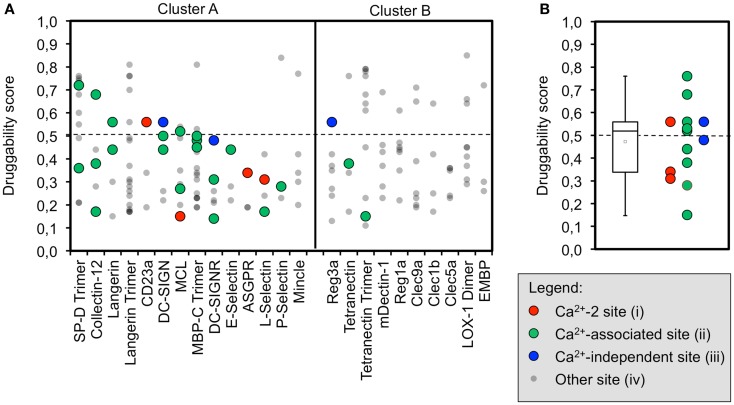
**Computational druggability prediction of CTLRs**. **(A)** A comprehensive account of all binding sites predicted by DoGSite is given. CTLRs were grouped according to the structure-based multiple sequence alignment. A druggability score of <0.5 is indicative of an undruggable binding site (dotted line). Binding sites are colored according to the corresponding category (red: Ca^2+^-2-binding sites (i), green: Ca^2+^-associated binding sites in long loop region (ii), blue: previously reported Ca^2+^-independent carbohydrate-binding sites (iii), gray: other binding sites). **(B)** A boxplot of the highest druggability scores of categories (i) to (iii) of each CTLR is shown. Tukey-style representation was chosen.

The initial identification of binding sites with DoGSite yielded between three and nine sites for CRDs and 9–19 for ECDs. Next, DoGSiteScorer was applied to calculate druggability scores. In the scoring scheme of this program, scores over 0.5 are indicative of a druggable binding site (73). At least one site that meets this criterion is found for the majority of the analyzed CTLRs (Figure 2A). However, targeting these sites with drug-like molecules will not necessarily exert an effect on the physiological function of the respective CTLR.

We propose that binding sites in proximity to Ca^2+^ ions located in the long loop region are relevant to carbohydrate recognition. Therefore, we assumed that small molecule-binding to these sites potentially modulates CTLR function. To this end, binding sites were assigned to four categories: (i) Ca^2+^-2-dependent, (ii) Ca^2+^-associated binding sites, (iii) Ca^2+^-independent carbohydrate-binding sites, and (iv) other binding sites (Figure 2A). Ca^2+^-associated binding sites (i, ii) were identified by DoGSite for all CTLRs coordinating a Ca^2+^-2 ion except for Mincle and the Langerin trimer. Experimentally determined Ca^2+^-independent carbohydrate-binding sites (iii) were identified for DC-SIGN, DC-SIGNR, and Reg3a (58, 82). The existence of a single druggable site is sufficient to render a target druggable. Accordingly, for each CTLR, sites assigned to categories (i) and (ii) displaying the highest score were selected for statistical analysis and a mean druggability score of 0.47 was calculated (Figure 2B). This classifies CTLRs as “difficult” or even “undruggable” targets (73). Notably, individual receptors such as SP-D and Collectin-12 possess favorable pockets in the long loop region. Other targets such as E-Selectin display druggability values well below the mean.

### Fragment screening reveals high hit rates against DC-SIGN, langerin, and MCL

**Figure 3:**
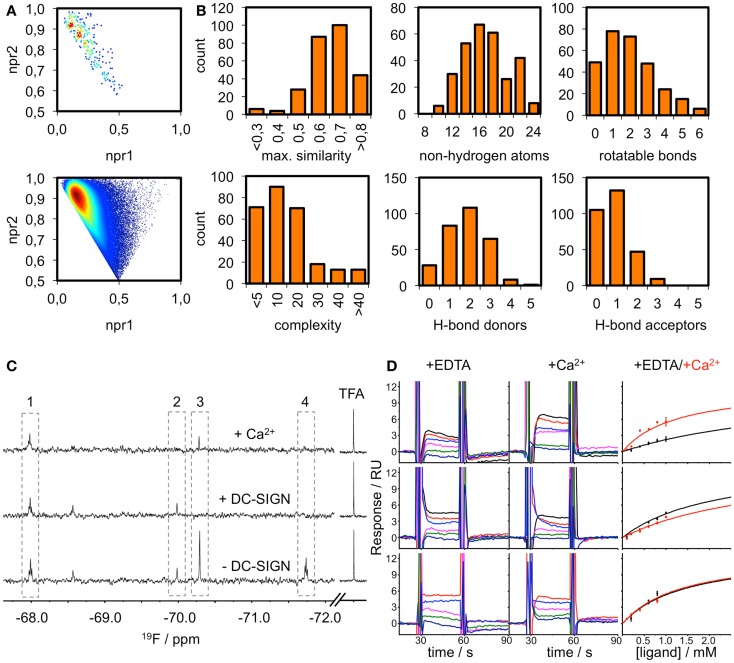
**Fragment library and screening against DC-SIGN**. **(A)** Molecular shape distribution of the 281 fluorinated fragments was assessed using normalized moments of inertia plots of the fragment library (top) and molecules from the ZINC database fragment set (bottom). **(B)** The diversity of the library is displayed for a selection of molecular descriptors. Histograms of the maximum pairwise similarity, number of non-hydrogen atoms, number of rotatable bonds, molecular complexity, and number of hydrogen bond donors/acceptors are shown. **(C)** Examples from the ^19^F NMR screen using T_2_-filtered spectra (*T* = 1.0 s, ν_CPMG_ = 50 Hz) showing compounds that do not bind (1), bind Ca^2+^-dependently (2), are competed by Ca^2+^ (3) and are binding at another binding site (4). **(D)** Sensorgrams for three examples from the SPR follow-up are shown. DC-SIGN ECD was immobilized on a CM7 chip. A one-site-binding model was used for fitting the data. This exemplifies compounds that experience increased (top), decreased (middle), or no alteration of affinity (bottom) in the presence of Ca^2+^. Data were extracted from regions of the sensorgram not perturbed by injection peaks. SPR sensorgrams are representatives of three independent measurements.

The existence of pockets on the surface of a receptor that are suitable to accommodate drug-like ligands can be assessed experimentally using fragment screening. The resulting hit rate serves as a predictor for druggability. Therefore, we composed a chemical library of fragments to be used in a homogeneous, label-free NMR-based screening assay. All fragments carry a fluorine atom, which allows for ^19^F NMR spectroscopy-based assessment of fragment binding. After quality control, 281 fragments were available for screening in 8 mixtures of maximum 36 fragments. The fragment library displays high shape and chemical diversity (Figures 3A,B).

DC-SIGN CRD and ECD were screened against the fragment library using ^19^F and T_2_-filtered ^19^F NMR spectra. Fragment binding to DC-SIGN was observed monitoring changes in chemical shift, line broadening, and T_2_ relaxation. Moreover, three spectra were recorded per fragment mixture. First, a spectrum was recorded in the absence of protein to exclude false positives such as Ca^2+^ chelators. The second spectrum was acquired in the presence of 10 μM protein to monitor fragment binding. Finally, Ca^2+^ was added in excess to the protein-fragment mixture, hypothesizing that metal binding to DC-SIGN modulates interaction of those fragments that are good candidates for inhibition of carbohydrate recognition (*vide supra*). Hits for DC-SIGN CRD and ECD were combined and frequent hitters were removed. Consequently, we identified 38 hits (13.5%) from mixtures binding to DC-SIGN in a Ca^2+^-dependent manner (Figure 3C). Out of these hits, 16 were found in both screenings and 21 hits were identified only during the CRD screening. Only one fragment was found while the ECD was used for screening.

**Figure 4:**
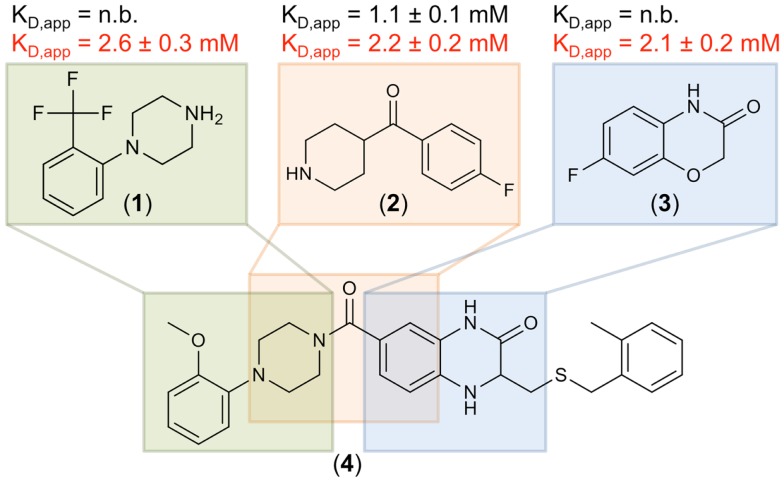
**Three fragment hits against DC-SIGN**. Fragments 1, 2, and 3 were identified during the primary ^19^F NMR screening and later confirmed in an SPR follow-up assay. These fragments are similar to substructures of a previously published micromolar DC-SIGN inhibitor 4 (41). Affinities measured in the presence of EDTA and Ca^2+^ are highlighted in black and red, respectively. No binding (n.b.) was detected in presence of EDTA for fragments 1 and 3. SPR measurements were run in triplicates for each condition.

To further validate these hits, SPR spectroscopy was employed as an orthogonal biophysical assay. This method not only detects binding of small molecules to macromolecules, but also allows for the determination of equilibrium dissociation constants. DC-SIGN ECD was immobilized on the chip surface and two experimental setups were utilized to differentiate Ca^2+^-mediated fragment binding from Ca^2+^-fragment competition. Fragments were injected either in the presence of 0.5 mM EDTA or 2 mM calcium chloride (Figure 3D), confirming a 1:1 binding model for 18 fragments (47%). Five fragments (13%) bound with a higher stoichiometry, 3 experienced no change in response in presence or absence of Ca^2+^ (8%), and 12 fragments (32%) did not give rise to detectable signals. The highest affinities measured were in the upper micromolar to lower millimolar range (0.6 mM < *K*_D,app_ > 1.3 mM). Of the 18 fragments confirmed by SPR, 9 showed increase in affinity upon Ca^2+^ addition and 9 displayed competitive behavior. Moreover, fragments similar to substructures of an already published submicromolar DC-SIGN inhibitor were identified (41, 42) (Figure 4). While fragments 1 and 2 bound competitive with the polysaccharide mannan in a ^19^F NMR competition assay, fragment 3 showed no such behavior upon addition of the natural carbohydrate ligand of DC-SIGN (data not shown).

In light of our computational analysis, we were surprised to find such a high fragment hit rate for DC-SIGN, and decided to expand our ^19^F NMR-based druggability prediction against the ECDs of two further CTLRs. We decided to screen our fragment library against Langerin being sufficiently distant to DC-SIGN in our structural sequence alignment (Figure 1A). To compare these findings to a CTLR more closely related to Langerin, we also included MCL in our analysis. Both proteins were expressed in *E. coli* and screened following the same protocol as for DC-SIGN. Again, Ca^2+^ was utilized as a competitor (Figures S5A,B in Supplementary Material) and several hits associated with Ca^2+^ binding were identified (Table 3). The pairwise overlap between the three CTLRs was low and none of the fragment hits bound to all CTLRs (Figure S5C in Supplementary Material).

**Table 3:** **Hit rates for three C-type lectin receptors from ^19^F NMR screening against a library of 281 compounds and hits confirmed by SPR for DC-SIGN**.

C-type lectin receptor	Ca^2+^-associated hits	Validated hits by SPR
DC-SIGN	38 (13.5%)	18 (6.4%)
Langerin	44 (15.7%)	
MCL	28 (10.0%)	

## Discussion

In this report, we assessed the potential of human CTLRs to be targeted with drug-like molecules. Therefore, we explored the ability of a set of CTLRs to accommodate inhibitors to modulate the receptor–carbohydrate interaction. This druggability prediction is an important part of the decision on whether a drug discovery campaign should be pursued (28–30). Despite a large body of recent research highlighting the importance of CTLRs in immune cell regulation, pathogen uptake, and as targets for adjuvants, only a few drug-like molecules have been developed for the CTLR family (2, 16, 17, 20). Herein, we aimed to rationalize why these receptors are considered challenging targets.

To start our investigations, CTLR druggability was predicted by computational methods. No data focusing on CTLRs are available and more general reports on glycan-binding proteins presented low druggability scores (39, 40). Unfortunately, the exact structures were either not disclosed or highly redundant and no CTLR was explicitly included. We assembled a set of 21 human CTLRs, and the murine Dectin-1. The latter was included as a reference as it is a well-studied CTLR and harbors a potential non-canonical, calcium-independent carbohydrate recognition site. The druggability prediction was performed using DoGSiteScorer, recently released software to predict the druggability of protein targets based on structural and physicochemical properties (73). Here, potential pockets on the protein surface were identified first, and then scored according to their physicochemical properties. Major determinants of druggability are depth, volume, and amino acid composition of the pocket (28, 32, 34, 36, 73). Generally, highly hydrophilic binding sites are considered undruggable (36).

Between three and nine binding sites were identified for CRDs, which is in accordance with values reported for other protein families (32). For Langerin, MBP-C, and Tetranectin, data on the homo-trimeric form were available. Here, the algorithm identified more potential sites, which is not surprising due to the larger surface area and symmetry of the assemblies (Figure 2A). Yet, targeting this initial set of binding sites does not necessarily interfere with carbohydrate recognition. Therefore, we categorized pockets according to their potential to modulate glycan binding. We argue that a druggable pocket located in close proximity of the long loop renders it a potential binding site for an inhibitor. The loop exhibits considerable movement in the absence of calcium as observed for other CRDs (65, 67, 83, 84) and adjacent sites have been proposed to communicate with the primary carbohydrate recognition site (22, 23). Four categories of sites were defined out of which only two, namely categories (i) and (ii), are either directly or indirectly associated with calcium ion binding.

The success-rate of detecting the canonical Ca^2+^-2 site (i) was low. Only 4 out of the 14 structures known to harbor such a site were identified (Figure 2A). This low number reflects a limitation of the employed pocket prediction, potentially due to shallow architecture of the Ca^2+^-2 sites. The low druggability score of the successfully identified Ca^2+^-2 sites corroborates this finding. Overall, these findings suggest that identification of carbohydrate recognition sites with computational algorithms such as DoGSite is challenging (*vide infra*).

Moreover, we analyzed a larger panel of sites associated with either the Ca^2+^-1, -2, or -3 site, summarized in category (ii) (Figure 2A). The criteria of this category were less stringent and based on an extended definition of sites potentially interfering with carbohydrate binding. Again, druggable sites were sparse. Collectin-12 and SP-D, both members of the Collectin group (CTLR group III), represent notable exceptions. Furthermore, our data on Langerin, for which monomer and trimer were analyzed side by side, highlight that subtle changes in the long loop region upon oligomerization abrogate the recognition of these sites by DoGSite (62).

Low scores for category (ii) sites are also found for members of cluster B of the sequence alignment. This cluster exclusively contains CTLRs not known to bind carbohydrates with their Ca^2+^-2 site (Figure 2A). The Ca^2+^-independent carbohydrate-binding sites of category (iii) found for Reg3a (group VII) is located in other regions of the CRD fold and has druggability scores of 0.56, predicting this CTLR to be challenging (82). Overall, only a few members of the CTLR family were predicted to be druggable (Figure 2B), which is in line with previous reports on glycan-binding proteins (39, 40).

To substantiate the computational studies, a ^19^F NMR-based fragment screening against one of the analyzed CTLRs was conducted. We chose DC-SIGN because as a viral uptake receptor it is of pharmacological interest and has been targeted in a high-throughput screening (41). While the successful HTS was already an indicator of DC-SIGN being amendable to fragment binding, the low druggability assessment by our computational analysis predicted a low hit rate of fragments interfering with any of the three DC-SIGN calcium sites. To our surprise, a high hit rate of 13.5% of the fragments from our library bound to DC-SIGN in Ca^2+^-associated sites during the NMR screening. The follow-up screening via SPR validated 18 (47%) of these fragments, a value not unusual for these two assay systems (85). Hits that were not validated by the SPR screening were either superstoichiometric binders (13%), not competitive with Ca^2+^ (8%), or had affinities below the detection limit of the SPR assay. The latter can be attributed to the high sensitivity of ^19^F NMR as a primary screen (38). Together, NMR and SPR result in a hit rate of 6.4%, which is in the expected range for fragment-based screenings and does not suggest a low likelihood to bind drug-like molecules (31, 37, 43, 86).

We performed the primary NMR screen against the CRD and the tetrameric ECD of DC-SIGN. Notably, only one fragment was uniquely identified during the screening of the ECD compared to 21 in the CRD screening. Conversely, many fragments binding to the ECD were later discovered to be false positives, such as frequent hitters from unrelated screening campaigns against non-CTLR targets. Hence, we conclude that screening for inhibitors has a lower false positive rate in absence of the neck region of DC-SIGN.

Another indicator for the validity of our screen to discover fragments inhibiting carbohydrate binding to DC-SIGN was the identification of the three fragments 1, 2, and 3. These hits are similar to substructures of the previously reported micromolar DC-SIGN inhibitor 4 (Figure 4) (41). In this respect, four has been shown to compete with carbohydrate binding and antagonized the DC-SIGN-mediated cell adhesion and particle uptake (41, 42). Direct competition between four and the three fragments was hampered by direct interaction of the fragments with four in absence of DC-SIGN (data not shown). Thus, mannan was employed to compete with fragments 1–3 and resulted in reproducible competition with fragments 1 and 2 (data not shown). Although, fragment 3 did not experience competition with the natural ligand, it can be speculated that it is associated with the binding site, as recognition was detected in SPR only in presence of Ca^2+^ (Figure 4). Moreover, other fragment hits showed even higher LE ranging from 0.30 to 0.37, which is a good starting point for further fragment evolution. A subsequent expansion of our ^19^F NMR-based screening to Langerin and MCL, also revealed similarly high hit rates (Table 3). Following up on these initial hits is subject of current research in the laboratory.

These encouraging experimental results are in contrast to our computational predictions. We attribute this conflict to the limitations of the DoGSiteScorer algorithm, which on the one hand is not parameterized for carbohydrate or metal binding sites (72) and on the other does not account for protein flexibility. Currently, there is no single software for druggability prediction available that is able to overcome these limitations.

Throughout the experimental evaluation, we employed competition with calcium ions as an indicator for the inhibition of carbohydrate recognition. We assumed the existence of allosteric sites originating from the flexibility of the long loop and cooperativity between the adjacent sites as previously described for other CTLRs (22, 23, 65, 67, 83, 84). In this context, it should be noted that accounting for conformational dynamics is recognized as a particular challenge for the development of improved algorithms (34).

To summarize, we report high *in silico* druggability scores for group III and V CTLRs as well as high experimental hit rates from fragment screenings against group II CTLRs. These data stand alongside with a successful drug design campaign that has already been launched against group IV CTLRs (19). Hence, we conclude that our data, while highlighting the limitations of current computational methods, support the assessment of CTLRs as suitable targets for drug-like molecules.

## Conflict of Interest Statement

The authors declare that the research was conducted in the absence of any commercial or financial relationships that could be construed as a potential conflict of interest.

## Supplementary Material

The Supplementary Material for this article can be found online at http://www.frontiersin.org/Journal/10.3389/fimmu.2014.00323/abstract
