# Supplementary material for: Computational and Experimental Prediction of Human C-Type Lectin Receptor Druggability
Source: Front Immunol. 2014 Jul 10;5:323. doi: 10.3389/fimmu.2014.00323 (PMC4090677; doi:10.3389/fimmu.2014.00323)
Supplement: Supplementary file 1 [file Data_Sheet1.PDF]

Supplementary Material

Computational and experimental prediction of human C-type lectin receptor druggability

Jonas Aretz[1,2], Eike C. Wamhoff[1,2], Jonas Hanske[1,2], Dario Heymann[1], Christoph Rademacher[1,2]*

[1]Department of Biomolecular Systems, Max Planck Institute of Colloids and Interfaces, 14424 Potsdam, Germany

[2]Department of Biology, Chemistry, and Pharmacy, Freie Universität Berlin, 14195 Berlin, Germany

***Correspondence:** Christoph Rademacher, Max Planck Institute of Colloids and Interfaces, Department of Biomolecular Systems, Am Mühlenberg 1, 14424 Potsdam.

Christoph.Rademacher@mpikg.mpg.de

1. Supplementary Data

Information on the pockets of category (i), (ii), and (iii) is available as a compressed .zip file. Folders contain the coordinates transformed according to the structural alignment and include pocket coordinates. Pocktes are numbered according to their scoring with the lowest number being highest score.

Nomenclature of the files:

Receptor: {CLTR name}/{PDB_ID}_receptor.pdb

Pockets: {CLTR name}/{PDB_ID}_pocket_{number}_{pocket category}.pdb

2. Supplementary Figures and Tables

2.1. Supplementary Tables

None.

2.2. Supplementary Figures

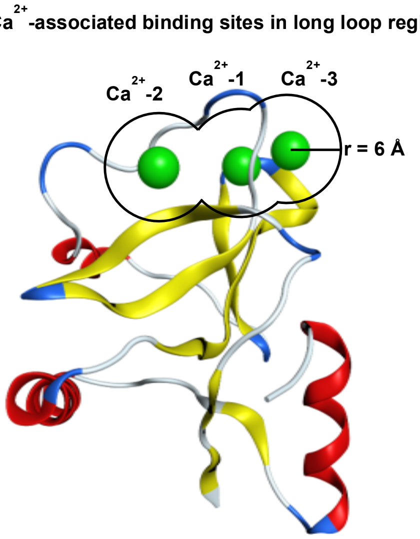

Supplementary Figure 1. Definition of binding site category (ii) for DC-SIGN. Predicted binding sites located within 6 Å of Ca^{2+}-1, -2 or -3 were assigned to category (ii) 'Ca^{2+}-associated binding sites in the long loop'.

```
1ESL        EAMTYDEASA YCQQ-R-YTH LVAIQNKEEI EYLNSIL--- SYSPSYYWIG
1G1S        -AYSWNISRK YCQN-R-YTD LVAIQNKNEI DYLNKVL--- PYYSSYYWIG
1H8U        --QTFSQAWF TCRRCY-RGN LVSIHNFNIN YRIQCSVS-- ALNQGQVWIG
1HUP        --MTFEKVKA LCVK-F-QAS VATPRNAAEN GAIQNLIK-- ----EEAFLG
1K9J        -QRNWHDSVT ACQE-V-RAQ LVVIKTAEEQ NFLQLQTS-- RSN-RFSWMG
1QDD        -RETWVDADL YCQN-MNSGN LVSVLTQAEG AFVASLIKES GTDDFNVWIG
1TN3        -TKTFHEASE DCIS-R-GGT LSTPQTGSEN DALYEYLRQS VGNEAEIWLG
1UV0        -PKSWTDADL ACQK-RPSGN LVSVLSGAEG SFVSSLVKSI GNSYSYVWIG
2C6U        -NLTWEESKQ YCTD-M-NAT LLKIDNRNIV EYIKARTH-- ----LIRWVG
2CL8        --NSWYGSKR HCSQ-L-GAH LLKIDNSKEF EFIESQTS-- SHRINAFWIG
2H2T        --KQWVHARY ACDD-M-EGQ LVSIHSPEEQ DFLTKRAS-- ---HTGSWIG
2OX8        -KEIFEDAKL FCED-K-SSH LVFINTREEQ QWIKKQM--- VGR-ESHWIG
2XR6        -QRNWHDSIT ACKE-V-GAQ LVVIKSAEEQ NFLQLQSSRS ---NRFTWMG
2YHF        -ESSWNESRD FCKG-K-GST LAIVNTPEKL KFLQDITD-- ---AEKYFIG
3CFW        -PMNWQRARR FCRDN--YTD LVAIQNKAEI EYLEKTL--- PFSRSYYWIG
3IKN        --KPFTEAQL LCTQ-A-GGQ LASPRSAAEN AALQQLVVAK ---NEAAFLS
3P5F        -PKTWYSAEQ FCVS-R-NSH LTSVTSESEQ EFLYKTAG-- ---GLIYWIG
3VPP        -WSIWHTSQE NCLK-E-GST LLQIESKEEM DFITGSLRK- IKGSYDYWVG
3WH3        -TKSWALSLK NCSAM--GAH LVVINSQEEQ EFLSYKK--- -PKMREFFIG
3WHD        -NKTWAESER NCSG-M-GAH LMTISTEAEQ NFIIQFL--- -DRRLSYFLG
1YPO        -SFNWEKSQE KCLS-L-DAK LLKINSTADL DFIQQAISY- -S-SFPFWMG

1DV8        LHDQN----- --GPWKWV-D GTDYETGFK- NWRP---EQP DDW-YGHGLG
1ESL        IRKVN----- --NVWVWVGT QKPLTEEAK- NWAP---GEP NNR-Q-----
1G1S        IRKNN----- --KTWTWVGT KKALTNEAE- NWAD---NEP NNKRN-----
1H8U        GRITGSGRC- --RRFQWVDG SRW---NFA- YWAA---HQP WSRG------
1HUP        ITDEKT--E- --GQFVDL-T GNRL--TYT- NWNE---GEP NNAGS-----
1K9J        LSDLNQ--E- --GTWQWV-D GSPLSPSFQR YWNS---GEP NNSG------
1QDD        LHDPKK--N- --RAWHWSSG SLV---SYK- SWGI---GAP SSVNP-----
1TN3        LNDMAA--E- --GTWVDMTG ARI---AYK- NWETEITAQP DGG-------
1UV0        LHDPTQGTEP NGEGWEWSSS DVM---NYF- AWERNPSTIS SP--------
2C6U        LSRQKSN--- --EVWKWE-D GSVISENMFE FLE-----DG -KG-------
2CL8        LSRN-QS--- -EGPWFWE-D GSAFF---PN SFQVRNAVPQ ESL-------
2H2T        LRNLDLK--- --GEFIWVD- GSHV--DYS- NWAPGE--PT SRS-------
2OX8        LTDSERE--- --NEWKWL-D GTSP--DYK- NWKA---GQP DNWGHG--HG
2XR6        LSDLNQE--- --GTWQWV-D GSPLLPSFKQ YWNRGE---P NNVG------
2YHF        LIYH-RE--- -EKRWRWI-N NSVFN---GN V-------TN QNQ-------
3CFW        IRKIG----- --GIWTWVGT NKSLTEEAE- NWGD---GEP NNKKN-----
3IKN        MTDSKTE--- --GKFTYP-T GESL--VYS- NWAP---GEP NDDGG-----
3P5F        LTKAGM---- -EGDWSWVDD TPFNKVQSAR FWIPGE---P NNAGN-----
3VPP        LSQDGH---- -SGRWLWQ-D GSSPSPGLL- ------PAER SQ-------S
3WH3        LSDQVV---- -EGQWQWV-D GTPLTKSLS- FWDVGEPNNI A---------
3WHD        LRDENA---- -KGQWRWVDQ TPFNPR-RV- FWHKNEP--- DNS-------
1YPO        LSRRNP---- -SYPWLWE-D GSPLMPHLFR ------VRGA VSQT-----Y

1DV8        GGEDCAHFTD -----D-GRW NDDVCQRPY- RWVCET-
1ESL        KDEDCVEIYI KREKDV-GMW NDERCSKKK- LALCY--
1G1S        -NEDCVEIYI KSPSAP-GKW NDEHCLKKK- HALCY--
1H8U        --GHCVALCT R----G-GYW RRAHCLRRL- PFICSY-
1HUP        -DEDCVLLLK -----N-GQW NDVPCSTSH- LAVCEFP
1K9J        -NEDCAEFS- -----G-SGW NDNRCDVDN- YWICKKP
1QDD        --GYCVSLT- -SSTGF-QKW KDVPCEDKF- SFVCKFK
1TN3        KTENCAVLSG ---AAN-GKW FDKRCRDQL- PYICQ--
1UV0        --GHCASLSR ---STAFLRW KDYNCNVRL- PYVCK--
2C6U        -NMNCAYFH- ------NGKM HPTFCENKH- YLMCER-
2CL8        -LHNCVWIHG -------SEV YNQICNTSS- YSICEK-
2H2T        QSEDCVMMRG -----S-GRW NDAFCDRKLG AWVCD--
2OX8        PGEDCAGLIY -----A-GQW NDFQCEDVN- NFICEKD
2XR6        -EEDCAEFSG -------NGW NDDKCNLAK- FWICKK-
2YHF        -NFNCATIGL ------TKTF DAASCDISY- RRICEK-
3CFW        -KEDCVEIYI KRNKDA-GKW NDDACHKLK- AALCY--
3IKN        -SEDCVEIFT -----N-GKW NDRACGEKR- LVVCEF-
3P5F        -NEHCGNIKA P----SLQAW NDAPCDKTF- LFICKRP
3VPP        ANQVCGYVKS -------NSL LSSNCDTWK- YFICEK-
3WH3        TLEDCATMR- -DSSNPRQNW NDVTCFLNY- FRICEM-
3WHD        QGENCVVLVY ---NQDKWAW NDVPCNFEA- SRICK--
1YPO        PSGTCAYIQR -------GAV YAENCILAA- FSICQK-
```

Supplementary Figure 2. Structure-based multiple sequence alignment of 22 CRDs. The PDB code of the analyzed structure is given in the first column.

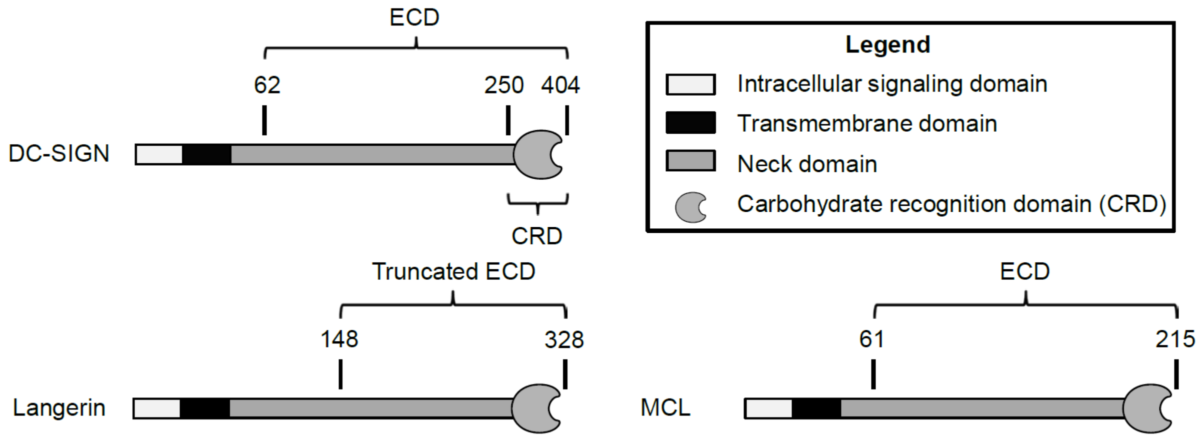

Supplementary Figure 3. Constructs of DC-SIGN, Langerin and MCL used in this study. Type II CTLRs consist of a carbohydrate recognition domain (CRD), a neck domain, a transmembrane domain and an intracellular signaling domain. Together, Neck domain and CRD, are referred to as extracellular domain (ECD). ECDs of the analyzed proteins were expressed as depicted. Additionally, the DC-SIGN CRD was expressed.

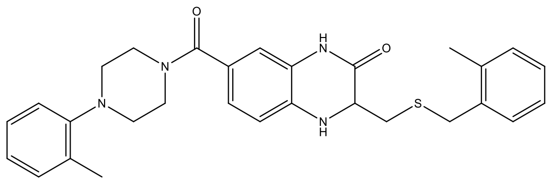

(**4**)

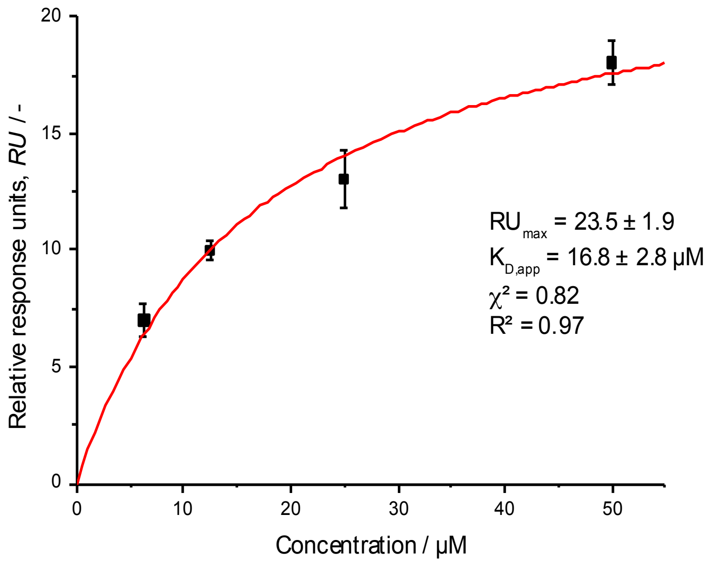

Supplementary Figure 4. Determination of RU_{max} of the CM7 chip used for SPR follow-up studies. Compound **4** (K781-9862, ChemDiv, San Diego, CA), which was identified previously in a HTS campaign against DC-SIGN, was dissolved in d_6-DMSO as 10 mM stock solution (Borrok and Kiessling, 2007). SPR analysis was performed in HBS-P + 2 mM calcium chloride using 180 s injection and 600 s dissociation time and a flow rate of 10 µL min^{-1} (c.f. Materials and Methods). As **4** was not soluble above 50 µM as measured by NMR and visible light spectrum, RU_{max} had to be determined by fitting using a 1:1 binding model (equation 1). Using this calculated value for RU_{max} and the molecular weight of **4**, the empirical factor A for protein activity was determined by transforming equation (2) to

$$A = \frac{RU_{max}}{RU_{immobilized}} \frac{MW_{protein}}{MW_{compound}}$$

yielding A = 0.6.

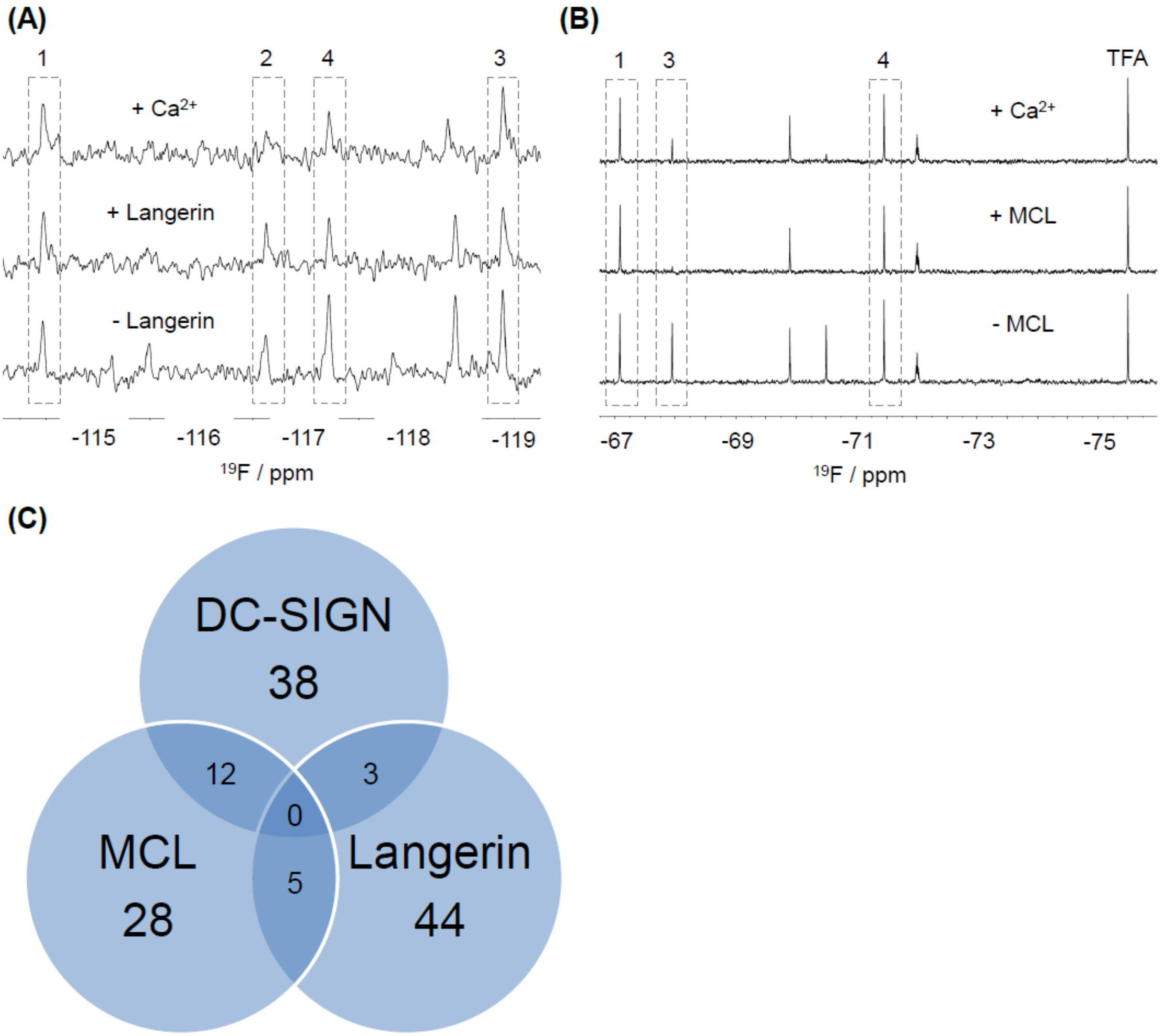

Supplementary Figure 5. NMR fragment screening against Langerin and MCL. (A, B) Example spectra from the ^{19}F NMR screen against Langerin (A) and MCL (B) using T_2 filtered spectra (T = 1 s, ν_{CPMG} = 50 Hz) showing compounds that do not bind (1), bind Ca^{2+}-dependently (2), are competed by Ca^{2+} (3) and are binding at another binding site (4). **(C)** Venn diagram depicting the overlapping fragment hits from NMR screening against DC-SIGN, MCL and Langerin.

3. References

Borrok, M.J., and Kiessling, L.L. (2007). Non-carbohydrate inhibitors of the lectin DC-SIGN. *J Am Chem Soc* 129**,** 12780-12785. doi: 10.1021/ja072944v.
